# Membrane Photobioreactor Applied for Municipal Wastewater Treatment at a High Solids Retention Time: Effects of Microalgae Decay on Treatment Performance and Biomass Properties

**DOI:** 10.3390/membranes12060564

**Published:** 2022-05-28

**Authors:** Hui Zou, Neema Christopher Rutta, Shilei Chen, Meijia Zhang, Hongjun Lin, Baoqiang Liao

**Affiliations:** 1 College of Geography and Environmental Sciences, Zhejiang Normal University, Jinhua 321004, China; zouzou@zjnu.edu.cn (H.Z.); neychriss@yahoo.com (N.C.R.); slchen@zjnu.edu.cn (S.C.); 2 Department of Chemical Engineering, Lakehead University, 955 Oliver Road, Thunder Bay, ON P7B 5E1, Canada; bliao@lakeheadu.ca

**Keywords:** membrane photobioreactor, microalgae decay, solids retention time, treatment performance, biomass properties, municipal wastewater treatment

## Abstract

Membrane photobioreactor (MPBR) technology is a microalgae-based system that can simultaneously realize nutrient recovery and microalgae cultivation in a single step. Current research is mainly focused on the operation of MPBR at a medium SRT. The operation of MPBR at a high SRT is rarely reported in MPBR studies. Therefore, this study conducted a submerged MPBR to treat synthetic municipal wastewater at a long solids retention time of 50 d. It was found that serious microalgae decay occurred on day 23. A series of characterizations, including the biomass concentration, chlorophyll-a content, nutrients removal, and physical-chemical properties of the microalgae, were conducted to evaluate how microalgae decay affects the treatment performance and biomass properties. The results showed that the biomass concentration and chlorophyll-a/MLSS dropped rapidly from 3.48 to 1.94 g/L and 34.56 to 10.71 mg/g, respectively, after the occurrence of decay. The effluent quality significantly deteriorated, corresponding to the total effluent nitrogen and total phosphorus concentration sharply rising and exceeding that of the feed. In addition, the particle became larger, the content of the extracellular polymeric substances (EPSs) decreased, and the soluble microbial products (SMPs) increased instantaneously. However, the filtration resistance had no significant increase because of the comprehensive interactions of the floc size, EPSs, and SMPs. The above results suggest that the MPBR system cannot maintain long-term operation under a high SRT for municipal wastewater treatment. In addition, the biological treatment performance of the MPBR deteriorated while the antifouling performance of the microalgae flocs improved after the occurrence of decay. The occurrence of microalgae decay was attributed to the double stresses from the light shading and intraspecific competition under high biomass concentration. Therefore, to avoid microalgae decay, periodic biomass removal is required to control the environmental stress within the tolerance range of the microalgae. Further studies are required to explore the underlying mechanism of the occurrence of decay.

## 1. Introduction

Wastewater reclamation and reuse have received more and more attention in the world as a result of the increasingly serious freshwater scarcity. At present, various wastewater treatment technologies, such as the activated sludge process (CAS) and membrane bioreactor (MBR), have matured and are widely used in practical applications [1,2,3,4,5,6]. However, most of these systems target organics removal using bacteria, and the treated effluent generally contains high levels of nitrogen and phosphorus. The direct disposal of such an effluent would cause eutrophication in the water body. Therefore, it generally requires additional processes targeting nutrient removal in order to meet discharge standards.

The microalgal membrane photobioreactor (MPBR), which integrates the photobioreactor (PBR) with the membrane filtration processes, is a promising technology for simultaneous microalgae cultivation and nutrient recovery [7,8,9,10]. For such a system, the use of sewage can offset the cost of the nutrients required for microalgae cultivation, and the microalgae biomass that is produced is one of the most promising precursors for biofuel production [11]. In addition, the greenhouse gas CO_2_ can be fixed by the microalgae through photosynthesis during the wastewater treatment process [12]. The feasibility of using MPBR for wastewater treatment has been extensively studied in the last decade [10,11,13,14,15,16,17,18,19].

As a complex biological system, the performance of MPBR is highly dependent on various operating conditions, such as lighting, hydraulic retention time (HRT), and solids retention time (SRT) [10,19]. Among all of these factors, SRT is a critical factor that has a significant influence on the biomass concentration, microalgal productivity, and nutrient removal in MPBR [15,18,20,21]. It is well known that a significant advantage of MPBR over PBR is the decoupling of HRT from SRT, which can reduce the downstream microalgal harvesting and dewatering due to the higher microalgae concentration that is achieved. However, researchers currently mainly adopt a medium SRT for the operation of MPBR [10,15,18,20]. To the best of our best knowledge, only two studies have operated MPBR at a long SRT [22,23]. For instance, Xu et al. [22] conducted MPBR at a prolonged SRT of 180 d for long-term operation, and eventually achieved a high biomass concentration of 4.84 g/L. A similar result was also reported by Praveen et al. [23]. However, the successes of these two studies were mainly attributed to the utilization of low organic strength secondary wastewater and a low initial microalgae concentration.

In fact, except for secondary wastewater, MPBR has also been applied for the treatment of high organic strength wastewater such as municipal wastewater and anaerobically digested wastewater [24,25]. However, the feasibility of the long-term operation of MPBR at a high SRT to treat municipal wastewater has never been reported. Therefore, a study on MPBR in this field is expected to provide valuable insight into the application of MPBR for municipal wastewater treatment.

In this study, a lab-scale MPBR system was operated at a high SRT of 50 d to explore the feasibility of long-term operation of the MPBR system under a high SRT for municipal wastewater treatment. A serious microalgae decay phenomenon occurred on day 23. The effects of microalgae decay on the treatment performance and biomass properties were then identified by a series of characterizations, including biomass production, chlorophyll-a concentration, nutrient removal, and microalgal properties. This study could provide practical experience for the operation and management of MPBR for high organic strength wastewater treatment.

## 2. Materials and Methods

### 2.1. MPBR Setup and Operation

**Figure 1:**
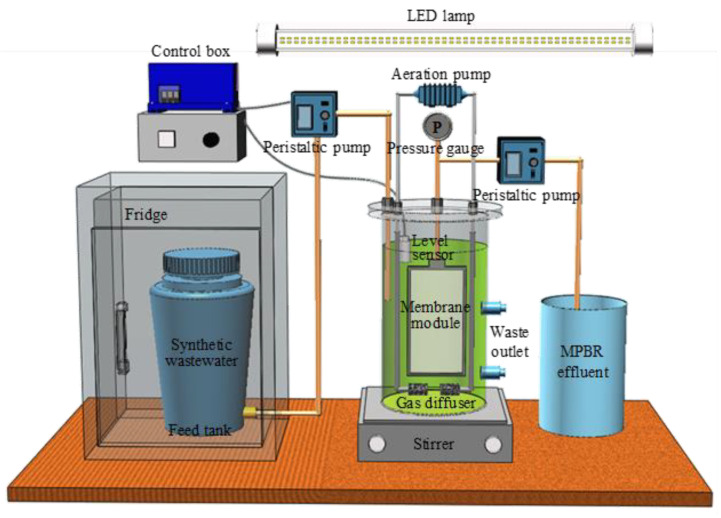
Experimental setup of the MPBR.

A lab-scale cylindrical submerged transparent MPBR system was conducted for municipal wastewater treatment. The schematic of this setup is displayed in Figure 1. Solid−liquid separation was accomplished using a flat plate membrane module. The membranes used in this work were commercial grade, and were purchased from SINAP Co. Ltd., Shanghai, China. Air was pumped into the reactor through an aeration pump to provide CO_2_ for microalgae growth and to form eddy currents to scour the membrane surface for fouling control. Gentle mixing was created using a magnetic stirrer (Model 6795-61, Corning, New York, NY, USA) located at the bottom of the reactor so as to prevent microalgal precipitation. Continuous illumination was provided by four LED lamps (two on each side). Details regarding the operating conditions and membrane module properties are listed in Table 1. *Chlorella vulgaris* (CPCC 90) that was precultivated in a modified salt medium (MSM) [26] was inoculated as the seed.

Simulated municipal wastewater was utilized as the feed in this study. The compositions of the synthetic influent are displayed in Table 2. The concentrations of glucose, nitrogen, and phosphorus were determined according to the corresponding concentration in the medium-strength municipal wastewater. The concentrations of trace elements were the same as those in the modified MSM medium for microalgae pre-cultivation. The feed was stored in a fridge at 5 °C and pumped by a peristaltic pump that was controlled by a level sensor (Madison Co., New York, USA). Another peristaltic pump was used to intermittently suck the permeate, using an operating mode of 3 min on and 2 min off.

### 2.2. Extraction and Analysis of Chlorophyll-a

The extraction and analysis of chlorophyll-a followed the method used by Nautiyal, Subramanian [27]. A known content of microalgae sediments was obtained by centrifugation at 8000× *g* for 10 min and was then resuspended into a certain volume of methanol. After that, the obtained microalgal suspension was immersed in a 60 °C water bath for 30 min and then cooled down to room temperature. The chlorophyll-a concentration in the solvent was spectrophotometrically determined using a visible spectrophotometer (DR2800, Hach) at three wavelengths. The content of chlorophyll-a in unit mass microalgae can be calculated using the following equation:

Chlorophyll-a/MLSS(mg/g) = (16.29(A^665.2^ − A^750^) − 8.54(A^652^ − A^750^))/MLSS (1)

where A^750^, A^665.2^, and A^652^ represent the absorbance at 750, 665.2, and 652 nm, respectively, and MLSS is the mixed liquor suspended solids of the microalgae.

### 2.3. PSD Analysis and Microscopic Observation

The PSD of the microalgae suspension was measured using a Malvern Mastersizer 2000 instrument (Worcestershire, West Midlands, UK) with a detection range of 0.02–2000 μm. Each sample was automatically measured in triplicate by the machine. This measurement was conducted one to two times per week.

The micromorphology of the microalgae was observed using an inverted optical microscope (Olympus IX51). For each sample, at least 30 images were randomly taken using a digital camera connected to the microscope.

### 2.4. Soluble Microbial Products (SMP) and Extracellular Polymeric Substances (EPS) Measurement

The SMP sample was collected from the microalgae suspension through centrifugation at 4000× *g* for 10 min and successive filtration through a 0.45 μm membrane. The bound EPS of the microalgae was extracted through a cation exchange resin (CER) (Dowex^TM^ Marathon^TM^ C, Na^+^ form, Sigma-Aldrich, Bellefonte, PA, USA) method [28]. Details regarding the extraction process can be found in our previous publication [29]. The total content of SMP and bound EPS were normalized as the sum of protein and carbohydrates, which can be determined colorimetrically according to Lowry’s method and Gaudy’s method, respectively [30,31].

### 2.5. Other Analysis

The pH and temperature of the suspension were measured using a pH meter (pH 700, Oakton, Charleston, SC, USA) and thermometer, respectively. The trans-membrane pressure (TMP) was monitored using a pressure gage. The growth of the microalgae was monitored through the determination of mixed liquor suspended solids (MLSS). The determination of MLSS was conducted following the standard method [32]. Total nitrogen (TN) and total phosphorus (TP) were measured following the methods previously adopted [26]. The analyses were conducted twice for each sample, and the average values were reported.

## 3. Results

### 3.1. Biomass Concentration and Chlorophyll-a Content

**Figure 2:**
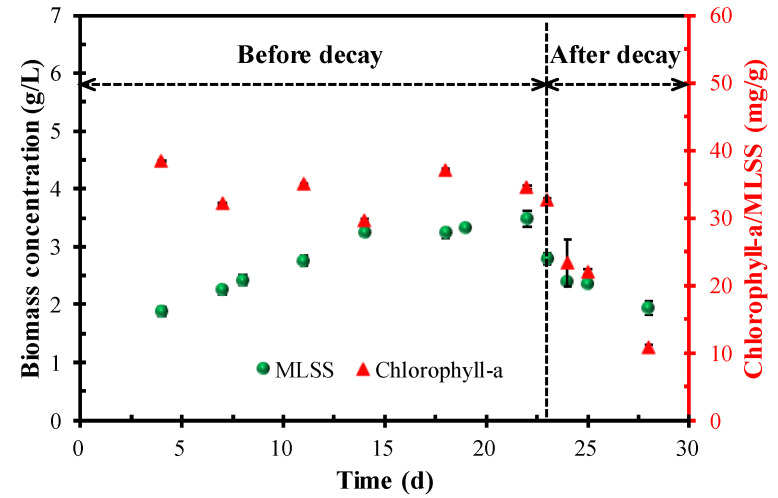
Variation in biomass concentration and chlorophyll-a/MLSS in the MPBR.

The contents of the microalgal biomass (represented by MLSS) and chlorophyll-a/MLSS are shown in Figure 2. During the period before the decay occurred, the microalgal biomass in the MPBR gradually increased and reached 3.48 g/L on day 22. Unlike the biomass concentration, the chlorophyll-a/MLSS content remained relatively stable and the average value was 34.44 ± 3.23 g/L. The gradual increase in microalgal biomass and relatively stable content of chlorophyll-a suggested that MPBR operated in a stable manner in the first 22 days. When the microalgae decayed on day 23, the biomass concentration and chlorophyll-a/MLSS dropped rapidly from 3.48 to 1.94 g/L and 34.56 to 10.71 mg/g, respectively, within 5 days. The rapid decrease in biomass concentration and chlorophyll-a/MLSS indicated that a large number of microalgae died in a short time.

The occurrence of microalgae decay suggested that a high SRT greatly impacted the continued long-term operation of MPBR. In this system, SRT directly affects the biomass concentration, which has a trade-off relationship with the effective light transmittance (i.e., a high biomass concentration corresponds to a low effective light transmittance) [15,17]. In the current work, a high SRT of 50 d was adopted, and the higher initial biomass concentration enabled the system to achieve a high MLSS of 3.48 g/L in a short time. With the increase in biomass concentration, the intraspecific competition among the microalgae became increasingly fierce because of the significant decrease in light transmittance. On the other hand, untreated municipal wastewater was used as the influent. Unlike the secondary effluent in previous studies, the organic matter in the municipal wastewater will lead to the growth of bacteria, which will enhance the stress effect on microalgae growth [33,34]. Because of the above two reasons, a large number of microalgae died, which led to a significant reduction in the biomass and chlorophyll-a/MLSS in the MPBR system.

### 3.2. Nutrients Removal

**Figure 3:**
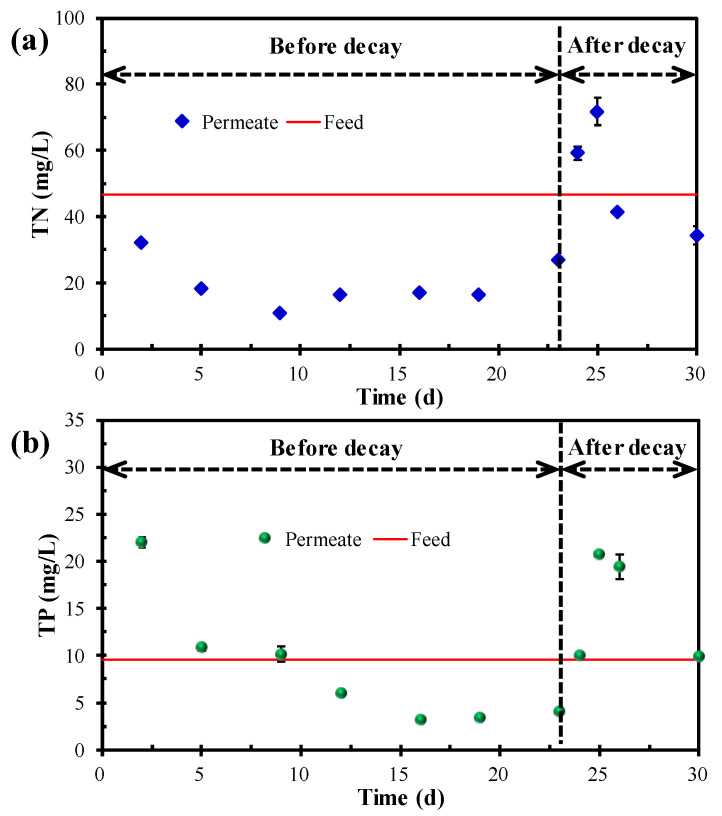
Variation in (**a**) TN and (**b**) TP in the feed and permeate.

Figure 3 shows the TN and TP concentrations in the feed and permeate. The real TN and TP in the feed were 46.7 ± 4.4 and 9.5 ± 0.4 mg/L, respectively. Under normal operating conditions (before the occurrence of decay), MPBR is a promising technique that can effectively remove TN and TP from the wastewater, although it requires a period of adaptation. In this study, the lowest TN and TP concentrations in the permeate were 10.9 and 3.2 mg/L, respectively, corresponding to the highest removal efficiency of 76.7% and 66.2%, respectively. However, once the decay occurred, both the TN and TP concentrations in the permeate significantly rose and even exceeded that of the feed. This result indicated that the released cytoplasm as a result of the microalgae decomposition would severely degrade the permeate quality. Although the microalgal decay phenomenon has seldom been studied in MPBR systems, such a phenomenon has been widely reported in natural systems such as lakes [35,36,37,38]. In addition, two days after the decay occurred, the TN and TP concentration in the permeate gradually decreased, suggesting that the destroyed MPBR system can self recover and the deteriorated permeate quality can also gradually improve. However, such a recovery process needs one week or even longer. Therefore, from the biological treatment performance, microalgae decay undoubtedly should be avoided and preferably prevented in advance in the practical operation and maintenance of MPBR.

### 3.3. Microalgae Properties and Membrane Fouling

#### 3.3.1. PSD and Micromorphology

**Figure 4:**
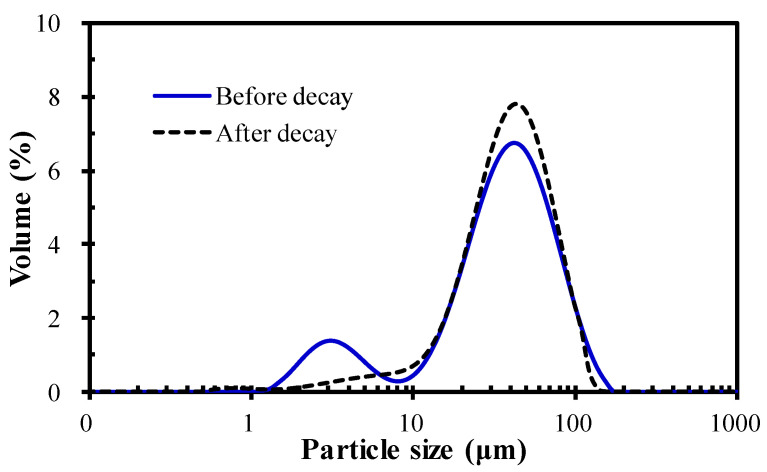
PSD of the microalgae in the MPBR before and after decay.

**Figure 5:**
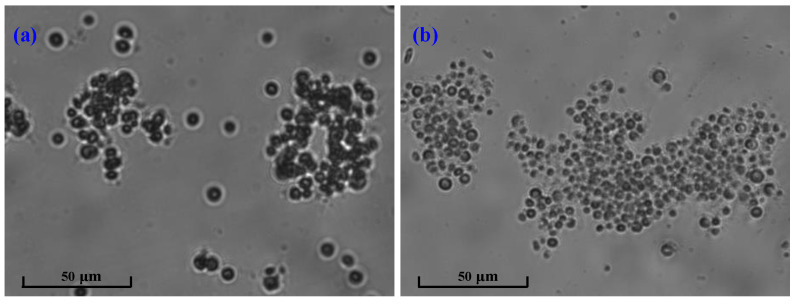
Microscopic morphology of the microalgae in the MPBR (**a**) before and (**b**) after decay.

Figure 4 shows the PSD of the microalgae suspension before and after the occurrence of decay. It can be seen that the suspended flocs before the occurrence of decay had a double-peak shape, corresponding to a sharp primary peak ranging from 10–100 µm and a weak secondary peak in the range of 1–10 µm. In comparison, the microalgae liquor after the occurrence of decay had a perfect unimodal shape; the peak in the range of 1–10 µm disappeared and the proportion of the flocs in the range of 10–100 µm increased. The microscopic morphology of the microalgae in Figure 5 further demonstrates the variation in PSD for the microalgae suspension before and after the occurrence of decay. As shown in Figure 5, *Chlorella vulgaris* cells dispersed individually or combined as flocs before the occurrence of decay, while almost existing as flocs after the occurrence of decay. The PSD and microscopic observation jointly proved that decay shock had a great influence on the biological properties of the microalgae particles, especially for the small dispersed *Chlorella vulgaris* cells. Combined with the sudden decline of biomass (Figure 2) and the surge of nutrients in the effluent (Figure 3), it can be reasonably speculated that the *Chlorella vulgaris* cells in the system might be the first to decompose under the stress environment, and the released substances from the lysis could promote the aggregation of free algal cells.

For the MPBR system, particle size is very important for membrane fouling formation. It is generally believed that larger size flocs have a lower fouling potential because of their lower adhesive ability and looser cake layer that can be formed [39,40]. From this aspect, it seems that the occurrence of decay favors membrane fouling control because of the improved floc size. However, it should be noted that microalgae decay also leads to the release of the cytoplasm, which will increase the type and content of pollutants in the system and then may aggravate membrane fouling [41,42]. Therefore, the final effect of microalgae decay on membrane fouling mainly depends on the comprehensive influence degree of the above two opposite factors (increased floc size and foulants content).

#### 3.3.2. EPS and SMP

**Figure 6:**
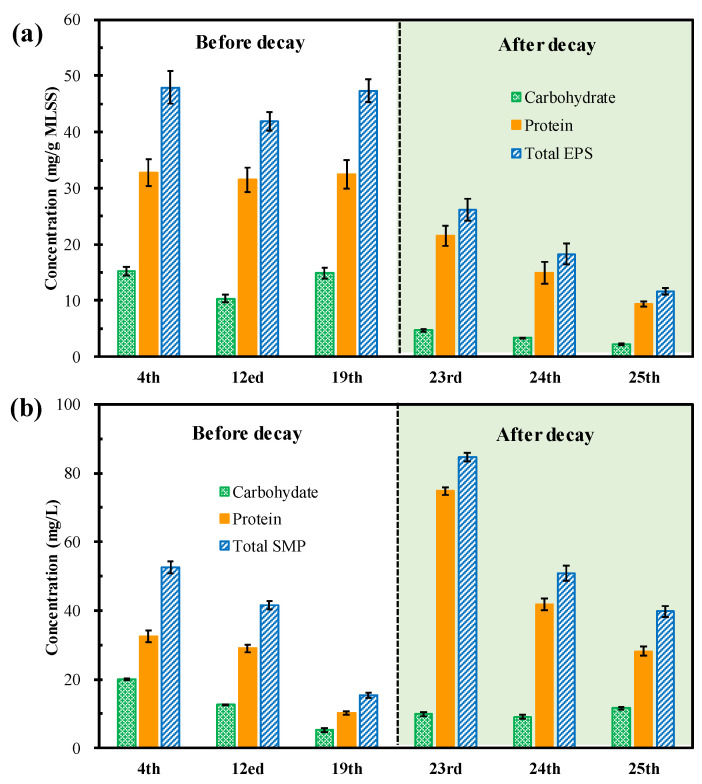
Comparison of the (**a**) EPS and (**b**) SMP content before and after decay.

Figure 6 compares the EPS and SMP values of the microalgal suspension before and after the occurrence of decay. It can be seen from Figure 6a that the amount of carbohydrates, proteins, and total EPS was comparable before the occurrence of decay, while gradually decreasing after the occurrence of decay. These results suggested that the microalgae flocs remained in a stable state before the occurrence of decay. However, after the decay occurred, a large number of microalgae cells died and decomposed suddenly, which led to the decomposition and corresponding decrease in EPS (from 26.18 ± 1.99 mg/g MLSS on day 23 to 11.61 ± 0.57 mg/g MLSS on day 25). Generally, EPS is considered a protective substance secreted by organisms to prevent them from being harmed in adverse environments [43,44]. As for membrane-related systems, a lower EPS is preferred because a higher EPS content would accelerate the formation of membrane fouling [45,46]. Therefore, according to the results obtained, it can be speculated that with the gradual increase in biomass concentration, the stress of photoinhibition and bacteria growth was too severe to be resisted by EPS secretion. On the other hand, it also suggests that the microalgae flocs after the occurrence of decay had better anti-fouling properties.

Figure 3b shows that the contents of total SMP gradually decreased from 52.63 ± 1.66 mg/L on day 4 to 15.41 ± 0.79 mg/L on day 19 (before the occurrence of decay), suddenly sharply increased to 84.68 ± 1.14 mg/L on day 23, and then gradually decreased to 39.75 ± 1.50 mg/L on day 25 (after the occurrence of decay). The variation trend in the protein was the same as that of the total SMP both before and after decay. SMPs are generally defined as biomass-released biopolymers and EPS hydrolysis is an important source of SMPs [45,47]. As illustrated above, the sudden increase in SMP corresponded to the decrease in EPS, reasonably demonstrating the outlet of the decreased EPS. SMPs also play a vital role in membrane fouling, especially when the main form of membrane fouling is gel layer formation, the increase in SMP content will significantly promote the increase in filtration resistance [42,48,49,50]. Therefore, after the occurrence of decay, the variation trends in EPS and SMP exhibited the opposite effects on membrane fouling. The comprehensive effects of EPS and SMP will rely on the main format of membrane fouling in the system.

#### 3.3.3. Membrane Fouling Performance

**Figure 7:**
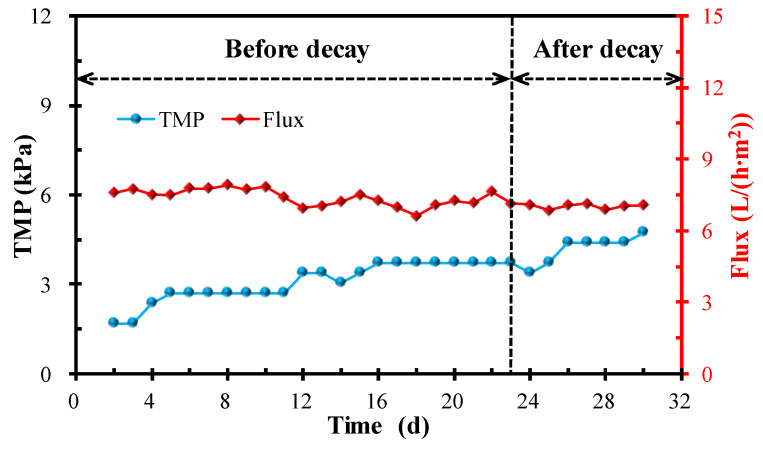
Variations in TMP and flux for the MPBR system.

The variations in TMP and flux for the MPBR system are displayed in Figure 7. It can be seen that the flux remained relatively constant and the TMP gradually increased with little fluctuation during the whole experimental period. The TMP increased from 1.69 kPa to 3.72 kPa before microalgae decay and then gradually reached 4.74 kPa after microalgae decay. Overall, there is no significant increase in TMP after the occurrence of decay. As stated above, the flocs size and EPS content exhibited the opposite effect when compared with the SMP content for the membrane fouling formation. The small rise in TMP further demonstrated that membrane fouling formation is the comprehensive interaction results of various factors.

### 3.4. Implications of High SRT for Long-Term Municipal Wastewater Treatment in MPBR

The occurrence of microalgae decay on the 23rd day suggests that the MPBR system cannot maintain long-term operation under a high SRT for municipal wastewater treatment. The occurrence of microalgae decay had a great impact on the MPBR system, which was reflected by the significant changes in the microalgae biomass, chlorophyll-a content, effluent quality, and the microalgae properties before and after the occurrence of decay.

Before the occurrence of decay, the microalgae biomass gradually increased, the chlorophyll-a and EPS content remained stable, and the TN and TP removal remained steady after a period of adaptation. The removal rate of TN and TP reached 10.54 and 2.14 mg/(L·d), respectively, which was comparable to or higher than that of most of the results that were previously reported [11,18,51]. From this aspect, it is feasible and promising to utilize microalgae for municipal wastewater treatment and microalgal biomass accumulation in the MPBR system under a high SRT.

However, the occurrence of microalgae decay on day 23 suggests that the above-mentioned feasibility is not always valid. That is, when the stress of the systematic environment caused by a high SRT exceeds the tolerance of the microalgae cells, the microalgae will start to die. In the current study, the high SRT and the application of municipal wastewater resulted in two main stresses on the microalgal cells. On the one hand, under high SRT operating conditions, the concentration of biomass gradually increased, which had a significant impact on light transmission. Ma et al. pointed out that all the light spectra attenuate exponentially with the light path based on a modified Cornet model for light transmission in the microalgal suspension [52]. In addition, the higher the microalgae concentration in the system, the faster the light attenuates at the same light path distance [52]. In this study, because of the separation effect of the membrane, the microalgae concentration (3.48 g/L) was very high compared with previous studies [14,18,22,53]. As a result, the photo masking effect was serious in the system. The severe insufficient light apparently will lead to fierce competition for light among the microalgae. On the other hand, unlike secondary effluents often reported in the literature, municipal wastewater has medium-strength organic matter, which provides a breeding ground for bacterial growth. In fact, the relationship between microalgae and bacteria is complex. They are cooperative and competitive [33,34]. Although suitable bacteria can provide CO_2_ for microalgae and thus facilitate the growth of microalgae, bacteria can also secrete toxic substances that limit the growth of microalgae [37]. Moreover, the growth of bacteria would preempt the growth space of microalgae and enhance the photo masking effect, which indirectly reduces the light transmission rate and enhances light competition among the microalgae. In this work, the microalgae started to die when the biomass content increased to 3.48 g/L. This indicated that the stress of the microalgal cells had reached the limit, which eventually led to the lysis of a large number of microalgal cells. Therefore, for MPBR systems operating at a high SRT, there should be a critical biomass concentration above which microalgae decay would occur. If the biomass can be effectively removed and always maintained below the critical value, then the total environmental stress can be controlled within the tolerance range of the microalgae, and the long-term stable operation can be maintained in the MPBR system.

After the decay occurred, the microalgae biomass, chlorophyll-a content, and nutrients removal decreased. From the perspective of biological performance, the occurrence of decay will not only reduce the biomass accumulation, but also reduce the effluent quality. The recovery of effluent quality required one week or even longer, and thus must be prevented in practice. Otherwise, the unsatisfactory effluents will enter the water body and cause problems such as eutrophication. On the other hand, from the perspective of membrane fouling, the particles became larger and the EPS content decreased, indicating that the microalgae flocs after decay had a better antifouling performance. However, the increased SMP suggested that more colloid-like substances were released into the system because of the occurrence of decay, which is beneficial to membrane fouling formation. As membrane fouling is the result of the comprehensive interactions of various factors, no significant TMP increase was observed after the occurrence of decay (Figure 7).

The above results provide some implications for municipal wastewater treatment and membrane fouling control in MPBR systems. According to the results in this work, a critical biomass concentration above which microalgae decay would occur exists. The MPBR system cannot maintain long-term operation under a high SRT for municipal wastewater treatment because the biomass concentration that can be achieved under a high SRT is too high. Therefore, to avoid microalgae decay, periodic biomass removal is required to control the environmental stress within the tolerance range of the microalgae. In addition, for MPBR, microalgae cells of a small size can more easily adhere to the membrane than sludge to form a filter cake layer, which will lead to serious membrane fouling problems [39]. Based on the above results, if a method or operating conditions can help flocculate free microalgal cells into flocs and prevent the increase in SMP content, the problem of membrane fouling can be significantly reduced. As a result, the subsequent enrichment and collection of microalgal biomass will also be facilitated because of the enlarged floc size.

It should be noted that, despite the valuable inspiration provided by this work, a distinctive limitation of this study is the design of a single experimental run. Although the single experimental run setting is not a special case and has been extensively applied in previous studies regarding MPBR [20,22,23], a duplicate design would be better and can avoid misinterpretation. In addition, too much biomass and bacterial development were speculated as to the potential reasons for microalgae decay. Apparently, the speculation was based on previous literature and the results obtained in this study. Nevertheless, their respective effects were not independently demonstrated in the current work. Therefore, further studies can be conducted on the following aspects in the future. For instance, finding the optimal HRT value by setting up experimental groups with HRT as a single variable. Afterwards, under the optimized HRT, several MPBRs can be operated in parallel with the long-term treatment of municipal wastewater. The effect of biomass concentration on the occurrence of decay can be confirmed by setting different SRTs. Furthermore, as organic matter could provide a breeding ground for bacterial growth, two types of wastewater, with and without organic matter, can be used as feed to verify the effects of bacterial development on the occurrence of decay.

## 4. Conclusions

In the current study, a lab-scale submerged MPBR was operated to treat synthetic municipal wastewater at a long SRT of 50 d. It was found that serious microalgae decay occurred on day 23, which had a great impact on the MPBR performance and the biological properties of the microalgae particles. A comparison of the microalgae properties showed that the biomass concentration, chlorophyll-a/MLSS, and effluent quality sharply decreased. However, the floc size increased, the EPSs content decreased, and the SMPs content increased. This suggests that the biological performance of the MPBR deteriorated while the antifouling performance of the microalgae flocs improved. However, the filtration resistance had no significant increase due to the comprehensive interactions of the floc size, EPSs, and SMPs. The occurrence of microalgae decay suggested that the MPBR system cannot maintain long-term operation under a high SRT for municipal wastewater treatment. The occurrence of decay was attributed to the double stresses from the light shading and intraspecific competition under a high biomass concentration. As a result, to avoid microalgae decay, periodic biomass removal is suggested to control the environmental stress within the tolerance range of the microalgae. Further studies are required in order to explore the underlying mechanism of the occurrence of decay in the future.

## Figures and Tables

**Table 1:** Operating conditions and membrane module properties of the MPBR system.

Parameters	Value
Working volume	9.64 L
Aeration rate	7.5 ± 0.03 L/min
Illumination intensity	8400 lux
SRT	50 d
HRT	2.9 ± 0.1 d
Operating temperature	25.2 ± 1.0 °C
Operating pH	6.81 ± 0.66
Membrane type	Flat sheet
Membrane material	Polyvinylidene fluoride (PVDF)
Effective surface area	0.03 m^2^
Pore size	0.1 μm
Membrane flux	7.30 ± 0.34 L/(h·m^2^)

**Table 2:** Composition of synthetic municipal wastewater.

Reagents	Element Concentration (mg/L)
Glucose	500
EDTA disodium salt dehydrate	64
NH_4_Cl	50 (N)
K_2_HPO_4_	3.55 (P)
KH_2_PO_4_	5.9 (P)
CaCl_2_·2H_2_O	3.0 (Ca)
MnCl_2_·4H_2_O	0.4 (Mn)
CoCl_2_·6H_2_O	0.1 (Co)
FeSO_4_·7H_2_O	1.0 (Fe)
Na_2_MoO_4_·2H_2_O	0.47 (Mo)
ZnSO_4_·7H_2_O	2.0 (Zn)
CuSO_4_·5H_2_O	0.4 (Cu)
H_3_BO_3_	2.0 (B)
MgSO_4_·7H_2_O	6.0 (Mg)

## Data Availability

Not applicable.
